# Prediabetes May Alter HPA Axis Activity and Regulation: A Study on Patients with Prediabetes

**DOI:** 10.3390/ijms26136231

**Published:** 2025-06-27

**Authors:** Palesa Mosili, Bongeka Cassandra Mkhize, Phikelelani Sethu Ngubane, Ntethelelo Hopewell Sibiya, Andile Khathi

**Affiliations:** 1 School of Laboratory Medicine and Medical Sciences, College of Health Sciences, University of KwaZulu-Natal, Westville 4000, South Africa; 215032519@stu.ukzn.ac.za (B.C.M.); ngubanep1@ukzn.ac.za (P.S.N.); 2 Pharmacology Division, Faculty of Pharmacy, Rhodes University, Grahamstown 6140, South Africa; n.sibiya@ru.ac.za

**Keywords:** HPA axis, prediabetes, cortisol, type 2 diabetes, stress response, epinephrine, depression, stress

## Abstract

A dysregulated hypothalamic–pituitary–adrenal (HPA) axis in patients with type 2 diabetes (T2D), a condition preceded by prediabetes, has been shown to exacerbate the hyperglycaemic state, increasing the risk of depression. However, HPA axis activity in a prediabetic state—as well as whether the prediabetic state affects HPA axis regulation—is not fully understood. This study investigated the activity of the HPA axis in selected biomarkers and hormones related to HPA axis regulation in individuals with prediabetes. The study used samples obtained from adults aged between 25 and 45 of all ethnicities from the King Edward VIII Hospital. The samples were divided into three groups—non-prediabetic (NPD) (*n* = 40), prediabetic (PD) (*n* = 40), and T2D (*n* = 40)—based on the participant’s glycated haemoglobin percentage. The cortisol (CORT), adrenocorticotropic hormone (ACTH), insulin, epinephrine (EPI), and norepinephrine (NE) concentrations of the samples were measured. The plasma CORT and ACTH concentrations in the PD group were higher compared to the NPD group. Plasma insulin concentration was increased only in the T2D group. There was also an increase in the plasma epinephrine concentration in the T2D group as compared to the NPD and PD groups. These observations collectively suggest that prediabetes is associated with heightened HPA axis activity and may alter HPA axis regulation, which may cause an altered stress response.

## 1. Introduction

The hypothalamic–pituitary–adrenal (HPA) axis is the central mediator of a highly integrated and intricately complex stress response often activated by various internal and external stressors [1]. The pathway’s main function is regulating the body’s response to stress [2,3]. Triggering of the HPA axis during a stress stimulus results in a cascade of stress hormone release, which disrupts the homeostatic state [4,5]. These hormones initiate a process of physiological changes by targeting different tissues—such as the skeletal muscles, adipose tissue, and liver—to mobilize great energy to counteract the stressor [6]. Cortisol (CORT), the end product and vital stress hormone of the HPA, plays a key role in glucose regulation and metabolism under basal conditions. Under stress conditions, its levels increase to raise blood glucose by targeting those different tissues to mobilize vast energy and prepare the body for action [4,7]. Once the energy supply is increased, CORT, along with components of the sympathetic–adreno–medullary (SAM) axis used in a regulatory feedback mechanism, stimulate different components of the HPA axis, decreasing CORT secretion and returning the body to a homeostatic “rest” state [4,6,8]. However, metabolic diseases, such as type 2 diabetes mellitus, has been shown to correlate with a dysregulated HPA axis and altered stress response, contributing to an increased risk of a mental health diagnoses [8,9,10].

Type 2 diabetes mellitus (T2DM) is characterized by a chronic hyperglycemic state, which is often attributed to insulin resistance in peripheral tissues, such as skeletal muscles and adipose tissue [11,12]. T2DM remains a major public health concern global, with the International Diabetes Federation (IDF) reporting 537 million adults aged 20–79 affected by diabetes in 2021 [13,14]. T2DM is often associated with numerous complications, including cardiovascular disease, kidney failure, and mental health disorders such as depression and anxiety [15]. Notably, various studies have associated alterations in hypothalamic–pituitary–adrenal (HPA) axis function with persons with diabetes, showing elevated CORT concentrations, impaired feedback mechanisms with evidence of dysregulated adrenocorticotropic hormone (ACTH) secretion, and imbalanced catecholamine levels—epinephrine (EPI) and norepinephrine (NE)—which may contribute to the development of depressive and anxiety symptoms [16,17,18,19,20]. However, it has not been established whether these changes associated with HPA axis dysregulation in T2DM are seen in the preceding state—prediabetes.

Prediabetes (PD) is characterized by moderate insulin resistance, where the blood glucose levels are above normoglycemic threshold yet below the diagnostic threshold for T2DM [13,21]. In 2021, it was reported that 541 million people worldwide were estimated to have prediabetes [14]. Although this state is often asymptomatic, recent studies have shown that complications seen in T2DM often begin during prediabetes [14,22,23,24]. An animal study conducted using a prediabetic model revealed a dysregulation of the HPA axis and the stress response [25]. However, these findings have not been investigated in individuals with prediabetes, and our study is among the first to explore this association under basal conditions.

The prevalence of prediabetes globally is high, and a recent review reported that the city of Durban, South Africa, had a 68% prevalence of prediabetes [26]. The review showed that the highest prevalence was found among individuals aged 25–45 years, which correlated with increased urbanization, sedentary lifestyles, high rates of hypertension, and obesity in this age group [22,26]. Another recent study reported hormonal imbalances that may be associated with prediabetes [27]. However, the alterations in the function of the HPA axis has not been studied in this population. Accordingly, this study investigated changes in the markers associated with the HPA axis in people with prediabetes in Durban, South Africa.

## 2. Results

This section presents a detailed analysis of the key biomarkers associated with HPA axis activity and its regulation, including cortisol (CORT), adrenocorticotropic hormone (ACTH), epinephrine (EPI), and norepinephrine (NE). The study population comprised three distinct groups—non-prediabetic (NPD), prediabetic (PD), and type 2 diabetic (T2D) individuals—with 40 participants enrolled in each group.

### 2.1. Population Demographics

The samples were profiled, and the demographics of the sample population are shown in Table 1. Chi-squared tests showed significant differences in age distribution *p* = 0.0441) and gender (*p* = 0.0481), with no significant difference in ethnicity (*p* = 0.8115) across the groups, indicating appropriate matching.

### 2.2. Glycated Hemoglobin, Plasma Glucose, and Plasma Insulin

**Figure 1:**
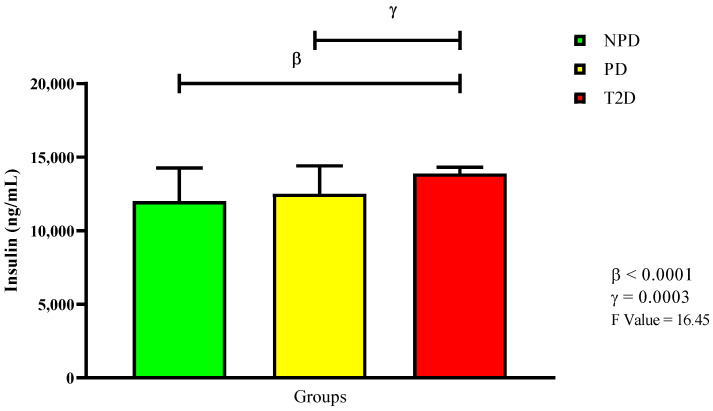
Insulin plasma concentration in non-prediabetic (NPD), prediabetic (PD), and type 2 diabetic (T2D) participants (*n* = 40, per group). Values are expressed as mean ± SD. β denotes comparison of NPD with T2D. γ denotes comparison of PD with T2D.

The samples were then divided according to their glycated hemoglobin (HbA_1c_) and fasting blood glucose (FBG) concentrations. Table 2 shows the extracted and analyzed median glycated hemoglobin concentrations and as percentages of the three groups, while the FBG concentration values are expressed in mean. Plasma insulin was measured, with Figure 1 displaying the measured insulin concentrations. The results show that there was no significant change in insulin concentration between the PD and NPD groups. Conversely, there was a significant increase in the insulin concentration in the T2D group compared to both the NPD (*p* < 0.0001) and PD groups (*p* = 0.0003).

### 2.3. Plasma Cortisol

**Figure 2:**
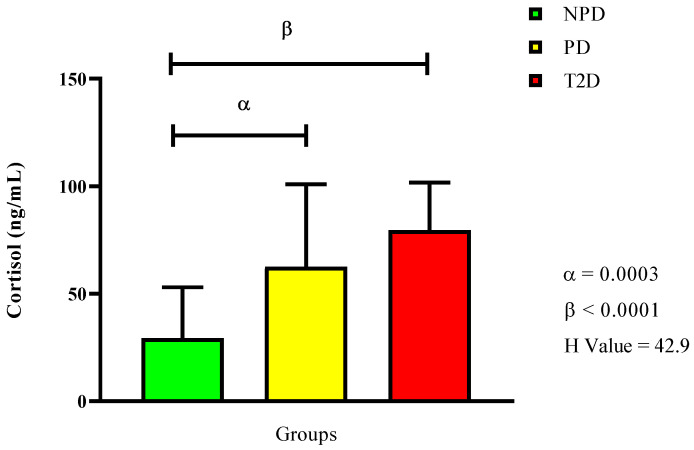
Cortisol plasma concentration in non-prediabetic (NPD), prediabetic (PD), and type 2 diabetic (T2D) participants (*n* = 40, per group). Values are expressed as follows: NPD median is 45.7 (11.5), PD median is 53.1 (19.7), and T2D median is 62.0 (10). α denotes comparison of PD with NPD. β denotes comparison of NPD with T2D. IQR (75–25th).

Figure 2 displays the plasma cortisol concentration, showing that both the PD and T2D groups had significantly higher levels compared to the NPD group (*p* = 0.0003 and *p* < 0.0001). However, there was no significan difference between the PD and T2D groups.

### 2.4. Plasma ACTH

**Figure 3:**
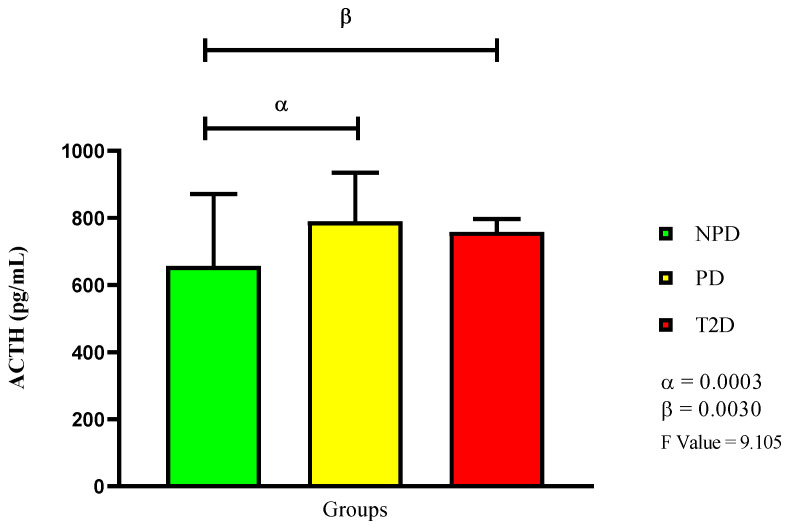
ACTH plasma concentration in non-prediabetic (NPD), prediabetic (PD), and type 2 diabetic (T2D) participants (*n* = 40, per group). Values are expressed as mean ± SD. α denotes comparison of NPD with PD. β denotes comparison of NPD with T2D.

In Figure 3, the results show that the PD group had a significantly higher plasma ACTH concentration compared to the NPD group (*p* = 0.0030). The T2D group also had a significantly higher concentration compared to the NPD (*p* = 0.0030), but a lower concentration than the PD group.

### 2.5. Plasma Epinephrine and Norepinephrine

**Figure 4:**
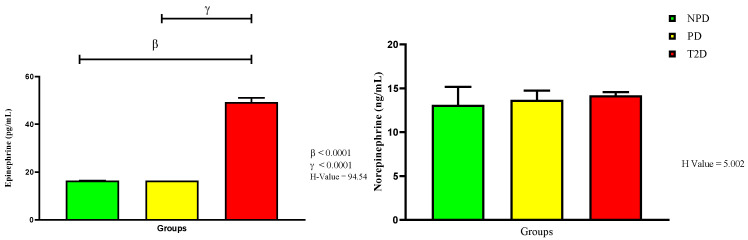
Epinephrine (EPI) and norepinephrine (NE) plasma concentrations in non-prediabetic (NPD), prediabetic (PD), and type 2 diabetic (T2D) participants (*n* = 40, per group). Values are expressed as follows: For EPI, NPD median is 1470 (612), PD median is 1284 (342), and T2D median is 49.3 (3.6). For NE, NPD median is 13.11 (3.79), PD median is 13.62 (5.08), and T2D median is 14.20 (1.05). IQR (75t–25th).

For this study, we further measured plasma catecholamines—epinephrine and norepinephrine—as presented in Figure 4. The results show that there were no significant changes between the NPD and PD groups. However, there was a significant increase in the epinephrine concentration in the T2D group compared to the NPD (*p* < 0.0001) and PD groups (*p* < 0.0001). The norepinephrine concentrations in all three groups were similar when each group was compared to the other.

## 3. Discussion

The HPA axis is a highly regulated pathway that plays a role in glucose homeostasis and is required for stress adaptation [4]. However, in T2DM patients, dysregulation of this pathway has been observed in numerous studies to contribute to the exacerbation of existing hyperglycemia and the aggravation of insulin resistance [19,28]. Furthermore, the heightened activity of the HPA axis—even under basal conditions—has been correlated with the increased risk and diagnosis of depression or anxiety [10]. Recent research has suggested that the complications of T2DM may begin during the preceding prediabetic stage, while animal studies have shown the changes that occur in the HPA axis in prediabetic models, but none of those findings have been verified in human studies [21,23,24,25]. In this study, we aimed to evaluate markers associated with HPA axis activity and its regulation in the prediabetic state in humans, comparing this group to non-prediabetic individuals and persons with T2DM in Durban, South Africa, where the prevalence of prediabetes is notably high [26].

The HPA axis responds to stress by initiating a hormonal cascade, culminating in the release of cortisol (CORT), the primary glucocorticoid involved in stress adaptation [3,29]. Cortisol plays a central role in the stress response by promoting gluconeogenesis in the liver and increasing energy availability in the peripheral tissues, which is used by different physiological processes and systems to respond to stress [30,31,32,33]. Once sufficient energy is produced, CORT levels are reduced through a negative feedback mechanism, returning the HPA axis to basal conditions [4]. However, in T2DM patients, it has been reported that CORT concentrations remain elevated, resulting in a persistently high basal concentration of CORT associated with the chronic hyperglycemia characteristic of T2DM [4,7]. In this study, it was confirmed that there was a significant increase in CORT concentration in the T2D group compared to the NPD group. Furthermore, the study observed a significant increase in CORT concentration in the PD group compared to the NPD group, while CORT concentration in the PD group was significantly lower than that in the T2D group. The elevated CORT concentration observed in the PD group could be caused by the altered peripheral regulation of this hormone. The peripheral regulation of CORT involves 11 beta-hydroxysteroid dehydrogenase type 1 (11β-HSD 1), which is highly expressed in insulin-sensitive tissues, such as the liver, muscle, and adipose tissue, converting inactive cortisone to active CORT [34,35,36]. Our results in the PD group suggest that elevated CORT levels may be due to increased 11β-HSD1 activity, particularly in the liver, where its expression is highest [35]. This upregulation may be associated with hyperglycemia, as the hyperglycemia-induced disruption of 11β-HSD1 has been shown to lead to HPA axis activation [35,37,38]. Further activation of the HPA axis results in increased CORT levels, which stimulate 11β-HSD1 expression in hepatocytes, myoblasts, and adipocytes, ultimately augmenting basal CORT concentrations as a compensatory response to the moderate hyperglycemia observed in PD [37,39].

The HPA axis, activated by stress, triggers the release of corticotrophin-releasing hormone (CRH) from the hypothalamus, which stimulates the anterior pituitary to secrete adrenocorticotropic hormone (ACTH) [40,41]. ACTH, once in systemic circulation, targets the adrenal cortex of the adrenal gland, resulting in CORT production and secretion [3,41]. ACTH is a crucial intermediary in this cascade, ensuring CORT production to maintain energy balance [2,4]. Once energy demands are met, ACTH secretion is reduced through a negative feedback mechanism, facilitating the return of the body to basal conditions by subsequentially reducing CORT production [4]. However, a majority of the literature reports that in T2DM, the negative feedback mechanism is impaired, resulting in dysregulated HPA axis activity, which is often evidenced by reduced ACTH concentrations; limited studies have reported increased ACTH alongside elevated CORT levels [7,19,42,43,44,45]. Contrary to most of the literature, the findings in this study showed increased ACTH concentrations in the T2D group compared to the NPD group. Additionally, the ACTH concentration in the PD group was significantly increased compared to NPD group, while it was significantly lower than that in the T2D group. ACTH is the primary regulator of CORT production and secretion [46]. Decreased ACTH concentration through the negative feedback mechanism leads to decreased GC secretion due to diminished stimulation of the adrenal cortex by ACTH [7]. However, the results of this study indicate that the hyperglycemia-induced increase in CORT levels may have reduced the sensitivity of the negative feedback mechanism, resulting in an irregular positive feedback mechanism in the PD group, as evidenced by the elevated ACTH concentration [25,46,47]. In addition, the possible increase in 11β-HSD1 activity—which may have contributed to increased CORT production in the PD group—could also have influenced HPA axis sensitivity, contributing to elevated basal ACTH levels observed in the study [37,39].

Various hormones including glucocorticoids (GCs) play a key role in glucose regulation and metabolism [3]. Acute GC exposure increases glucose concentration by enhancing hepatic gluconeogenesis via the upregulation of phosphoenolpyruvate, opposes insulin action by inhibiting glucose uptake in muscles, and amplifies adipose tissue lipolysis [8]. Furthermore, during acute glucocorticoid-induced insulin resistance, pancreatic cells compensate by increasing insulin secretion in non-diseased individuals, which triggers the insulin signaling pathway in insulin-dependent tissues such as the skeletal muscle, resulting in glucose uptake and promoting glycogen synthesis [2,8]. As glucose is taken up and circulating levels decrease, insulin secretion is inhibited [2]. However, in T2DM, altered HPA axis function, chronic stress, and persistently high glucose concentrations dysregulate glucose metabolism, contributing to prolonged exposure to GCs. This results in persistently elevated glucose concentration and prolonged glucocorticoid-induced insulin resistance, which also progressively reduces insulin sensitivity and eventually contributes to sustained hyperglycemia and hyperinsulinemia [34,48,49]. Our results confirm previous studies, showing significantly elevated HbA1c and fasting blood glucose levels in the T2DM group compared to the NPD group, indicating chronic hyperglycemia. This is further supported by elevated insulin levels in the T2D group compared to NPD group, suggesting insulin resistance, as the high glucose concentrations reflect defects in insulin secretion, action, or both. Similarly, the PD group had elevated glycated hemoglobin and plasma glucose levels compared to the NPD group, but the results were significantly lower than the T2DM group. However, insulin levels in the PD group were similar to those in the NPD group and significantly lower than those in the T2D group.

PD pathophysiology is often ascribed to the decline in glucose metabolism regulation, the decreased sensitivity of insulin in insulin-dependent tissues, and the subsequent dysfunction of pancreatic β-cells, resulting in hyperglycemia and the overall dysregulation of insulin [23,24]. In the presence of elevated CORT, as seen in our PD group results, gluconeogenesis in the liver may be upregulated, increasing endogenous production of glucose in the blood and potentially contributing to the intermediate hyperglycemic state [50]. Furthermore, elevated GCs antagonize insulin action by directly inhibiting pancreatic-β cells from secreting insulin, impairing glucose uptake in various insulin-dependent tissues—such as by inhibiting the translocation of the glucose transporter GLUT4—and interrupting insulin signaling, which may have further aggravated the hyperglycemic state in the PD group [2,24,51]. Although there was no increase in insulin concentration in the PD group, this may imply that the baseline increase in CORT was not long-term but sustained long enough to contribute to the elevated glucose levels observed [49,52]. The intermediate hyperglycemic state in the PD group—which may be attributed to the elevated CORT levels—may also alter the SAM axis, which in turn regulates HPA axis function.

Catecholamines—epinephrine (EPI) and norepinephrine (NE)—are released from the adrenal medulla as part of the sympathetic–adreno–medullary (SAM) axis, which, together with the HPA axis, forms the integrated stress response system [53,54]. Under basal conditions, circulating catecholamines remain low, as most are reabsorbed or metabolized locally, playing a role in the regulation of glucose homeostasis by modulating the HPA axis [55,56,57]. However, during an acute stress stimulus, there is a significant increase in plasma NE and EPI levels, which promotes glycogenolysis and gluconeogenesis, raising blood glucose to meet increased energy demands, while also stimulating the HPA axis [4,7]. However, hyperglycemia, as seen in T2DM, has been shown to induce cellular stress and activate intracellular signaling pathways that impair adrenergic receptor function in the adrenal medulla, thereby dysregulating catecholamine production [56]. This dysregulation leads to elevated epinephrine (EPI) levels [56]. Additionally, altered EPI and norepinephrine (NE) secretion in T2DM disrupts glucose homeostasis, with studies showing that irregular catecholamine release driven by impaired adrenergic signaling results in prolonged EPI secretion and impaired feedback regulation [56,58,59]. Consequently, this contributes to increased glucose production and reduced insulin sensitivity, exacerbating hyperglycemia in T2DM [58,59]. Elevated EPI levels in the T2D group compared to the NPD group confirmed previous research. However, NE concentration of the T2D group remained similar to the NPD group. Furthermore, the current study observed that EPI concentration in the PD group was significantly lower than in the T2D group, but comparable to the NPD group. NE concentration in the PD group was similar to both the NPD and T2D groups. These results suggest a potential early disruption of the sympathetic–adrenal–medullary (SAM) axis in the PD group, as the HPA axis plays a pivotal role in modulating this process.

The dysregulation of catecholamines activates the HPA axis and increases GC secretion, which exacerbates insulin resistance and disrupts glucose utilization, contributing to the hyperglycemic condition observed in the PD group [58,60,61]. Dysregulated EPI release, which may stem from impaired receptor sensitivity or disrupted synthesis pathways, may disrupt the HPA axis feedback mechanism, leading to increased ACTH and CORT levels, as observed in the PD group results [55,58,62,63,64]. This imbalance, along with altered EPI regulation, fosters a neurobiological milieu characterized by altered neurotransmitter dynamics and mood regulation, as well as reduced synaptic plasticity, thereby increasing the risk of depression [65]. Furthermore, prolonged hyperglycemia triggers intricate signaling cascades within adrenal chromaffin cells, upregulating NE secretion as a compensatory mechanism to restore HPA axis equilibrium [66]. Resultant elevated NE influences changes in the adrenergic receptors in the hypothalamus, pituitary, and adrenal gland, modulating CRH and ACTH release and acting as a regulatory mechanism to counterbalance the effects of hyperglycemia [64,67,68,69,70]. However, the consistent NE levels across all groups may reflect an impaired feedback mechanism, contributing to the EPI dysregulation observed in the T2DM group and the corresponding hyperglycemic state in PD group.

## 4. Materials and Methods

### 4.1. Study Design and Setting

This study was a quantitative cross-sectional study using stored blood samples obtained from patients at the King Edward VIII Hospital (KEH) in Durban, South Africa. The study was reviewed and approved by the University of KwaZulu-Natal Biomedical Research Ethics Committee (BREC) (Ref no. BE266/19) and the Provincial Health Research Committee from KZN Department of Health. All included blood samples were collected in EDTA tubes and tested for fasting blood glucose concentrations in the mornings—between 07:00 and 09:00—following overnight fasting. Samples were drawn from undiagnosed male and female individuals aged 25–45, of diverse ethnicities, to ensure similar circadian rhythm and ensure accurate blood glucose concentration measurement. Blood samples excluded individuals with a history of liver disease, kidney disease, adrenal gland diseases, heart disease, depression, and anxiety. Furthermore, individuals who were heavy smokers and alcohol drinkers were excluded. Samples from pregnant women and professional athletes were also excluded from the study. Informed consent was provided by all participants.

### 4.2. Sample Size Determination

To determine the sample size, Gpower software 3.1.9.4 was used. A sample size sufficient for a power of 0.80, with a confidence interval of 95% and a margin of error of 5%, was targeted and achieved. For reliable statistical comparison across the groups, this resulted in each group comprising a total of 40 samples.

### 4.3. Sampling

The 120 samples of undiagnosed patients were divided based on the fasting blood glucose (FBG) levels into the following three groups: non-prediabetic (NPD) (*n* = 40), prediabetic (PD) (*n* = 40), and type 2 diabetic (T2D). Prediabetes was determined based on glycated hemoglobin (HbA_1c_) concentrations of 39–46 mmol/mol (5.7–6.4%) and fasting blood glucose (FBG) concentrations of 5.6–6.9 mmol/L, in accordance with the American Diabetes Association (ADA) criteria [13]. T2D was determined using a glycated hemoglobin (HbA_1c_) concentration of 48 mmol/mol (6.5%) and an FBG concentration of 7.0 mmol/L and above, according to the same criteria. HbA_1c_ was measured at the KEH using NGSP- and IFCC-certified, laboratory-based Tosoh G8 HPLC Analyzer. The blood was centrifuged (Eppendorf 5403, Hamburg, Germany) at 4 °C, 10,000× *g* for 15 min. Plasma was collected and stored at −80 °C in a Bio Ultra freezer (Snijers Scientific, Assendelft, The Netherlands) until further biochemical analysis.

### 4.4. Biochemical Analysis

Insulin and cortisol (CORT) were determined in the plasma samples using the Human Metabolic Hormone Magnetic Bead Panel protocol from the MILLIPLEX^®^ MAP Kit (Merck, Darmstadt, Germany), according to the manufacturer’s instructions. The concentrations were measured using the Bio-plex MAGPIX Multiplex Reader (Bio-Rad Laboratories Inc., Hercules, CA, USA) and quantified using the Bio-Plex Manager version 6.1 software. Plasma adrenocorticotropic hormone (ACTH), epinephrine (EPI), norepinephrine (NE) concentrations were measured using their respective enzyme-linked immunosorbent assay (ELISA) kits (Elabscience Biotechnology Co., Ltd., Wuhan, China), according to the manufacturer’s instructions.

### 4.5. Statistical Analysis

All statistical analyses were performed using GraphPad Prism version 8.0.2 software (GraphPad Software Inc., San Diego, CA, USA). Chi-squared tests were used to assess group comparability of the population demographics. The Shapiro–Wilk test was used to assess the normal distribution of the data. For parametric data, differences between the means of the independent non-prediabetic (NPD), prediabetic (PD), and type 2 diabetic (T2D) groups were assessed using one-way analysis of variance (ANOVA), followed by Tukey’s post hoc test, and reported as mean ± standard deviation (SD). The Kruskal–Wallis test was used to assess non-parametric data, followed by Dunn’s multiple comparison test, and the data were presented as median and interquartile range (IQR). A *p*-value of <0.05 was considered as statistically significant.

## 5. Conclusions

This study’s observations highlight the potential intricate interplay between the HPA axis and the adrenal-derived catecholamines, along with their downstream effects on glucose regulation and metabolism. This relationship may underlie the complex pathophysiological mechanisms contributing to the intermediate hyperglycemia seen in this study and, ultimately, the development of prediabetes. Based on the basal conditions of the parameters assessed, it may be inferred that there is an association between prediabetes and heightened HPA axis activity, which may contribute to further hyperglycemia and altered HPA axis regulation. This, in turn, may indicate an altered stress response and an increased risk of depression and progression to type 2 diabetes. To our knowledge, this is the first study to report on HPA/SAM axis dysregulation in persons with prediabetes. Although the sample size was limited, these preliminary findings underscore the need for further research. Future investigations involving larger, more diverse cohorts and incorporating factors such as lifestyle, diet, and perceived stress levels are essential to fully elucidate the role of HPA axis in prediabetes. In view of the influence of HPA hyperactivity on worsening dysglycemia, this study supports the potential value of targeting HPA axis regulation in prediabetes as a strategy to prevent or delay conversion into overt diabetes.

### 5.1. Study Limitations

While this study was able to identify an association with prediabetes and altered HPA axis activity, it had several limitations. Measuring the complete characteristics of the HPA axis in humans within a clinical setting remained challenging due to sample type restrictions and overall study time constraints. Dynamic assessments of cortisol (CORT) and adrenocorticotropic hormone (ACTH)—such as the dexamethasone suppression test or the corticotropin-releasing hormone (CRH) stimulation test—could not be conducted. Additionally, the lack of heparinized plasma, which would have been more optimal for catecholamine analysis, limited the ability to draw definitive conclusions about negative feedback dysregulation in the HPA axis. Furthermore, although the study discussed the potential link between HPA axis dysregulation and the increased risk of depression, the retrospective design of the study prevented the use of psychiatric assessment tools—such as the PHQ-9 or structured clinical interviews—making it difficult to accurately assess associations with depressive symptoms.

### 5.2. Future Studies Recommendations

Future studies should incorporate dynamic assessments of HPA axis components over extended periods and in alternative sample types, such as saliva or urine. The inclusion of urinary catecholamine measurements would enhance the understanding of SAM axis involvement. A larger and more diverse sample size, with comprehensive data on variables such as BMI, medication use, comorbidities, and disease duration, would strengthen findings. Longitudinal follow-up studies would also be valuable to better understand stress physiology in prediabetes and T2DM. Furthermore, incorporating psychiatric evaluation tools and collaborating with mental health professionals could help explore the relationship between chronic stress, HPA axis activation, and prediabetes. The emerging literature supports the exploration of pharmacological interventions targeting both depression and glucose metabolism, with a recent meta-analysis showing that agomelatine—a melatonergic antidepressant—may improve both glycemic control and mood symptoms in patients with type 2 diabetes [71]. Another study showed that pharmacological regulators of the HPA axis—such as cabergoline and metyrapone—have positive outcomes on fasting blood glucose [72] and can serve as promising therapeutic agents for managing both depressive symptoms and glucose metabolism in patients with T2DM and prediabetes. Additionally, early interventions—whether pharmacological or non-pharmacological—targeting the HPA/SAM axis should be further explored. Notably, insulin treatment has been shown to normalize HPA axis function in diabetic patients, although similar effects on the SAM axis were not observed [73].

## Figures and Tables

**Table 1:** Demographics of sample population.

	Non-Prediabetes (*n* = 40)	Prediabetes (*n* = 40)	Type 2 Diabetes (*n* = 40)	X^2^ (df)	*p*
**Age (*N* = 120)**				6.242 (2)	0.0441
25–35	11	13	4		
36–45	29	27	36		
**Gender (*N* = 120)**				6.07 (2)	0.0481
Male	15	20	26		
Female	25	20	14		
**Ethnicity (*N* = 120)**				2.98 (6)	0.8115
African	25	24	28		
White	7	4	3		
Indian	8	10	8		
Coloured	0	2	1		

**Table 2:** Glycated hemoglobin, fasting blood glucose (FBG) concentration, and plasma insulin concentration in non-prediabetic (NPD), prediabetic (PD) and type 2 diabetic (T2D) participants (*n* = 40 per group). HbA1c values are expressed as median (75–25th **^#^**), and β denotes comparison of NPD with T2D (*p* < 0.0001). γ denotes comparison of PD with T2D (*p* < 0.0001). FBG values are expressed as mean ± SD.

Groups	HbA1c (mmol/mol)	HbA_1c_ %	FBG (mmol/L)
**NPD**	36 (4 ^#^) ^β^	5.4 (0.3 ^#^) ^β^	5.7 ± 0.24 ^β^
**PD**	43 (3 ^#^) ^γ^	6.1 (0.3 ^#^) ^γ^	6.6 ± 0.38 ^γ^
**T2D**	75.5 (33.25 ^#^)	9.3 (4.2 ^#^)	11.95 ± 3.39

^β^ *p* < 0.0001denotes comparison of NPD with T2D; ^γ^ *p* < 0.0001 denotes comparison of PD with T2D. **^#^** IQR(InterQuartile Range) (75–25th).

## Data Availability

The datasets generated during the current study are available from the corresponding author on reasonable request.
